# WNT/β-Catenin Signalling and Epithelial Patterning in the Homoscleromorph Sponge *Oscarella*

**DOI:** 10.1371/journal.pone.0005823

**Published:** 2009-06-08

**Authors:** Pascal Lapébie, Eve Gazave, Alexander Ereskovsky, Romain Derelle, Chantal Bézac, Emmanuelle Renard, Evelyn Houliston, Carole Borchiellini

**Affiliations:** 1 Centre d'Océanologie de Marseille, Aix-Marseille Université, CNRS - UMR 6540, Station marine d'Endoume, Marseille, France; 2 Faculty of Biology and Soils, Saint-Petersburg State University, St. Petersburg, Russia; 3 Université Pierre et Marie Curie-Paris 6, CNRS UMR 7009 Biologie du Développement, Observatoire Océanologique, Villefranche-sur-Mer, France; 4 Centre d'Océanologie de Marseille, Aix-Marseille Université, CNRS–UMS 2196, Station marine d'Endoume, Marseille, France; Ecole Normale Supérieure de Lyon, France

## Abstract

Sponges branch basally in the metazoan phylogenetic tree and are thus well positioned to provide insights into the evolution of mechanisms controlling animal development, likely to remain active in adult sponges. Of the four sponge clades, the Homoscleromorpha are of particular interest as they alone show the “true” epithelial organization seen in other metazoan phyla (the Eumetazoa). We have examined the deployment in sponges of Wnt signalling pathway components, since this pathway is an important regulator of many developmental patterning processes. We identified a reduced repertoire of three divergent *Wnt* ligand genes in the recently-sequenced *Amphimedon queenslandica* (demosponge) genome and two *Wnts* from our EST collection from the homoscleromorph *Oscarella lobularis*, along with well-conserved genes for intracellular pathway components (β-catenin, GSK3β). Remarkably, the two *O. lobularis Wnt* genes showed complementary expression patterns in relation to the evenly spaced ostia (canal openings) of the exopinacoderm (ectoderm), highly reminiscent of *Wnt* expression during skin appendage formation in vertebrates. Furthermore, experimental activation of the Wnt/β-catenin pathway using GSK3β inhibitors provoked formation of ectopic ostia, as has been shown for epithelial appendages in Eumetazoa. We thus suggest that deployment of Wnt signalling is a common and perhaps ancient feature of metazoan epithelial patterning and morphogenesis.

## Introduction

The earliest steps in animal evolution remain obscure, but can be illuminated by comparative studies between the most basally branching animal groups, notably sponges, cnidarians and ctenophores. The sponges are widely accepted as one of the oldest metazoan lineages, with the unicellular choanoflagellates forming a sister group to the metazoa as a whole [1], [2], [3], [4]. Since sponge choanocytes and choanoflagellates show many similarities, the first step of animal evolution has been proposed to have been the acquisition of multicellularity from a choanoflagellate-like ancestor, with early metazoans comprising epithelial-type cell layer containing feeding choanocytes and non-feeding cells [1], [5], [6]. Such multicellular epithelia are considered as a fundamental metazoan innovation, enabling a range of morphogenetic processes and leading to body plan diversification [7].

Among the four sponge lineages -Hexactinellida, Calcispongiae, Demospongiae and Homoscleromorpha- [8], [9] the homoscleromorphs are a key group for understanding the origin and evolution of epithelia [1], [10]. The homoscleromorphs are the only sponge group to possess eumetazoan-like true epithelia, characterized by the presence of basement membrane with type IV collagen and regularly distributed *zonula adhaerens* cell junctions both in larval and in adult forms [11], [12], [13]. In addition, unlike other sponges, they show epithelial-type morphogenesis during development [14], [15]. In this context, it is interesting to note that the Homoscleromorpha and the Eumetazoa share quasi equivalent molecular toolkits for two key aspects of epithelial function, cell adhesion and signalling [16]. A subset of molecules involved in both these functions has been identified in Choanoflagellates, and therefore predates multicellularity [17], [18].

Among major signalling molecules, the WNTs are metazoan protein ligands that control diverse processes such as cell proliferation, cell-fate determination, cell migration and differentiation during multiple steps of embryonic development [19], as well as in tissue homeostasis in adults, regulating stem cell populations [20] in regenerating tissues including Hydra polyps and [21] vertebrate intestine [22]. The WNT family appears to have made an early apparition and diversification during animal evolution, since twelve of the thirteen known *Wnt* subfamilies have been identified in cnidarians [23] but none in the genome of the choanoflagellate *Monosiga brevicollis* [18].

WNT ligands can influence cell behaviour by activating a number of distinguishable downstream intracellular pathways [24]. The most intensively studied is the “canonical” or Wnt/β-catenin pathway, in which WNT binding to Frizzled family receptors provokes stabilization and nuclear translocation of the transcriptional co-regulator β-catenin via inhibition of the cytoplasmic kinase GSK-3β [19]. Components of this pathway have been identified in Demospongiae [25], with polarized expression of a *Wnt* gene in *Amphimedon queenslandica* larva suggesting a role in embryo patterning [26].

Among the Homoscleromorpha, the existence of Wnt signalling components was noted previously in *Oscarella carmela* [16]. We now report complementary expressions of two *Wnts* in adult tissue of *Oscarella lobularis* [10]. By comparison with known epithelial *Wnt* expression patterns in Eumetazoa, the observed expression patterns indicate that canonical Wnt signalling is involved in epithelial patterning in these animals. This conclusion was supported by our pharmacological inhibition experiments, the first functional test of the role of Wnt signalling in a sponge. In the light of these findings we propose that Wnt signalling have a common and perhaps ancestral function in metazoans to regulate epithelial morphogenesis,

## Results and Discussion

### Low WNT complexity in basally diverging metazoan phyla

**Figure 1:**
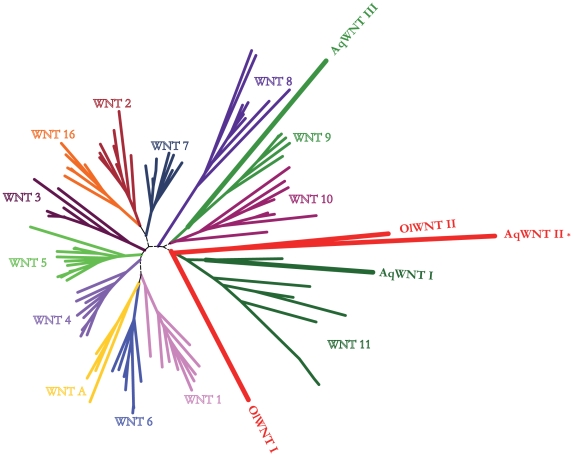
Maximum likelihood inference tree of the WNT families. Sponge branches are bold: OlWNT-I, -II (*Oscarella lobularis*) and AqWNT-I, -II and –III (*Amphimedon queenslandica*). AqWNT-II (asterisk) corresponds to the published Amphimedon sequence [26]. Bootstrap support values and Bayesian posterior probabilities are available in the Supplementary file S1.

There has been no previous exhaustive survey of *Wnt* representation in sponge genomic data. We identified three *Wnt* sequences in the complete genome sequence of *Amphimedon queenslandica* (*AqWNTI-III*), as well as two from an *Oscarella lobularis* EST collection (*OlWNTI-II*). The *Wnt* gene repertoire in *A. queenslandica* is thus markedly less extensive than that of cnidarians and bilaterians, as it has been found for other developmental gene families [27]. All five predicted sponge WNT proteins showed typical structural characteristics, including a highly conserved cysteine pattern (alignment available upon request) and peptide secretion signals, but their sequences were highly divergent compared to a reference dataset comprising the complete WNT repertoire retrieved from selected deuterostome, protostome and cnidarian genomes. They could thus not be assigned orthology to any of the previously defined eumetazoan WNT sub-families (Figure 1), as has been the case for WNTs from fast-evolving bilaterian [28] and hydrozoan [23], [29] species. Another indicator of the considerable evolutionary divergence of WNT sequences was the sensitivity of the relationships between WNT subfamilies to the method of analysis and to gene sampling. Given this marked divergence of *Wnt* sequences, more genomic data from different sponge clades and from other basally diverging metazoan phyla will be required to discriminate between the evolutionary scenarios of *Wnt* gene loss in one or more sponge groups from a larger set present in the last common ancestor of Metazoa, *versus* significant diversification of the family in the eumetazoan lineage post-dating the split between eumetazoans and sponges.

### Complementary *Wnt* expression patterns in the homoscleromorph exopinacoderm

**Figure 2:**
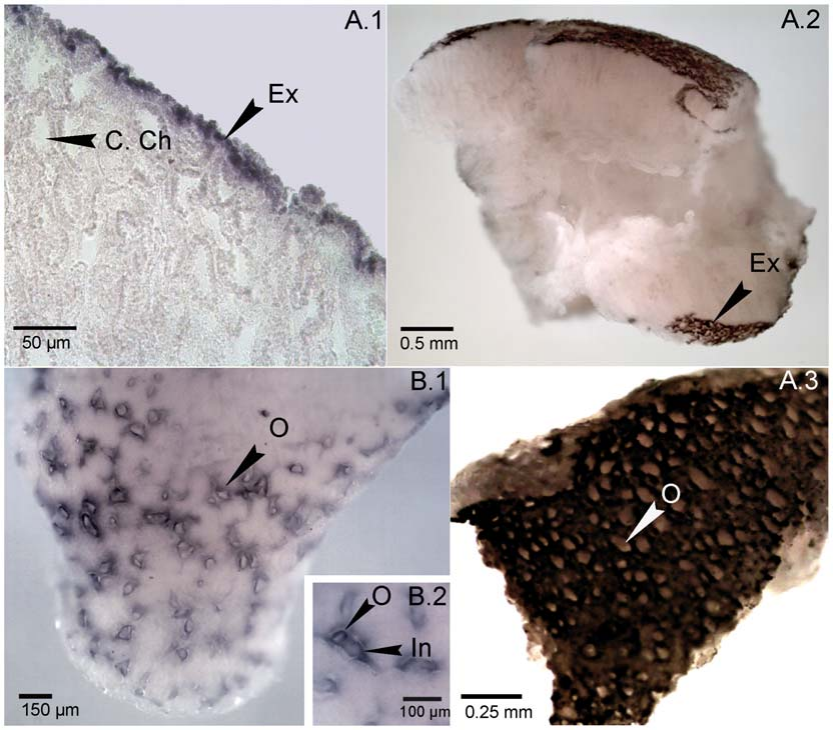
Complementary expression patterns of *Wnt* ligands in *Oscarella lobularis* exopinacoderm. *In situ* hybridization on serial histological sections (A.1) and whole mount sponge pieces (A.2–3, B.1–2) with digoxygenin RNA probes for *OlWnt-I* (A.1–3) and *OlWnt-II* (B.1–2). A.1–3: *OlWnt-I* mRNA expression localizes exclusively to the inter-ostial exopinacoderm. B: *OlWnt-II* signal is specifically elevated in ostia. (B.2) Higher-magnification view of an ostium and inhalant channel. (B.1, A.3). Ch. C: Choanocyte chamber. Ex: Exopinacoderm. In. C:Inhalant channel. O: Ostium. Positive and negative controls for these techniques under the same conditions have been performed and gived same results as previously on both whole mounted and sectioned material [5].

**Figure 3:**
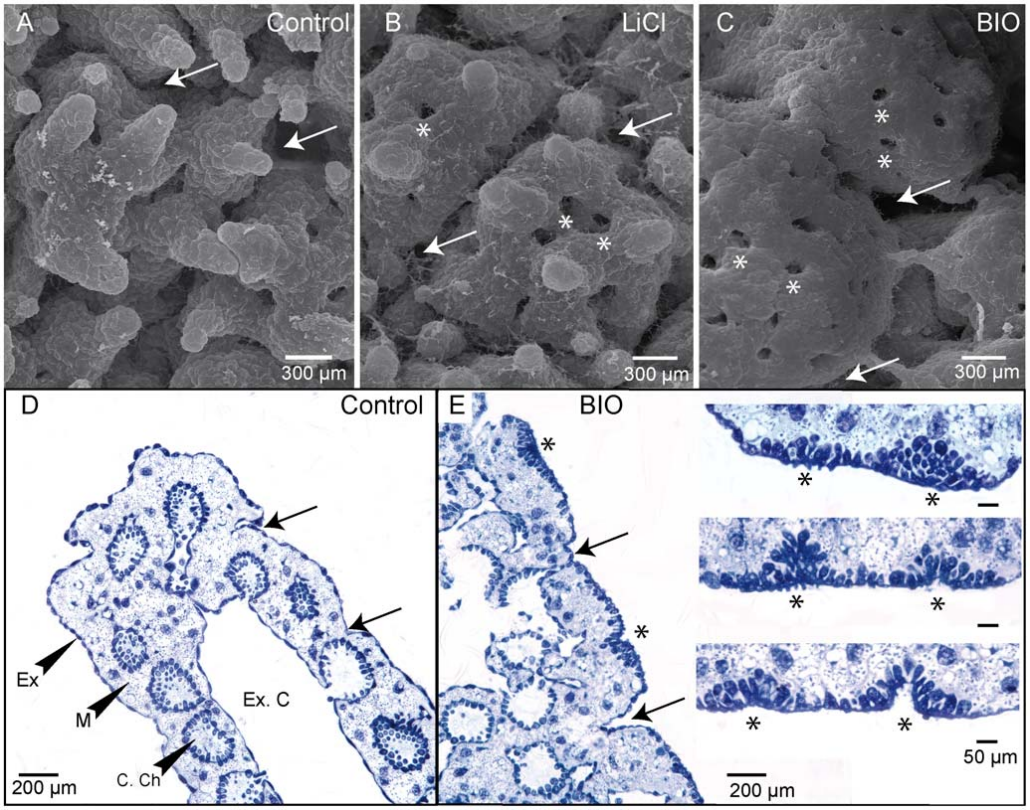
Stimulation of Wnt/β-catenin signalling promotes ostia formation. A–C: Scanning electron microscope images of pieces of sponge cultured without treatment (A), treated for 72 h with 40 mM LiCl (B), or treated for 48 h with 0.5 µM BIO (C). Both LiCl-treated and BIO-treated samples have more ostia than controls. Ostia pre-existing before treatment are shown by arrows and ectopic ostia by asterisks. D–E: Semi-thin sections of untreated (D) and BIO-treated (E) sponge explants, showing changes in epithelium morphology. Note increased number and ovoid shape of epithelial cells in treated samples. Higher-magnification views of sequential sections of ectopic ostia forming by epithelial invagination from placode-like structures are shown on the right panels in E. Similar results were obtained with LiCl-treated sponges (data not shown). Ostia existing before treatment are shown by arrows and ectopic ostia are shown by asterisks. (Ch. C) Choanocyte chamber. (Ex) Exopinacodem. (Ex. C) Exhalant channel. (M) Mesohyl

Expression patterns of the two *Oscarella Wnt* genes, determined using fixed pieces and serial sections of adult sponges, revealed striking complementary expression domains in the outer epithelial cell layer, the exopinacoderm (Figure 2). *OlWntII* expression (Figure 2B1–B2) was detected exclusively in entry zones (ostia) of the inhalant channels, which are regularly spaced and formed by epithelial invagination of the exopinacoderm (Figure 3E). Similar localized *Wnt* expression has been described during epithelial appendage formation in diverse eumetazoan species, including *Hydra* and *Nematostella* tentacles [23] and the *Drosophila* wing [30].

In contrast to *OlWntII*, *OlWntI* RNA was detected in the intervening regions of the exopinacoderm (Figure 2A1–A3). The complementary expression patterns of the two *O. lobularis Wnts* are thus strongly reminiscent of *Wnt* expression during the ectodermal morphogenesis in vertebrates.. In vertebrates, specific WNT ligands (WNTs 10-b, 1, 3a,) activate the formation of spaced epithelial appendages such as hairs, mammary glands , taste papillae, teeth and feathers [31]. Distinct *Wnts* (*Wnt11* for feathers [31], *Wnt3* and *Wnt7-b* for teeth [32], *Wnt4* and *Wnt6* for hair [33]) are expressed in the inter–appendage epithelium. *OlWntI* expression in the inter-ostial exopinacoderm is clearly comparable with the inter-appendage *Wnt* expression in vertebrate skin, and *OlWntII* expression in ostia with *Wnts* expressed in developing skin appendages. We cannot rule out the possibility that the phenomena of complementary *Wnt* expression in epithelial layers is more widespread, since the lack of similar descriptions protostomes, invertebrate deuterostomes other non-bliaterians may result from a general lack of attention to *Wnt* expression in adult epithelia.

The similarity of the complementary *Wnt* expression patterns in *Oscarella* and vertebrate epithelial appendages clearly should not be taken as evidence for homology of these structures, particularly given their late appearance during ontogeny and the phylogenetic distance between the groups. Thus any parallelism between *Wnt* expression in the exopinacoderm and eumetazoan epithelial layers such as skin probably results from evolutive convergence, favoured by the inherent patterning properties of WNT signalling systems.

### Activation of the Wnt/β-catenin pathway promotes ectopic ostia formation

In vertebrate models it has been shown that the WNT ligands implicated in activating skin appendage formation via the canonical pathway [32], [34], [35], [36], [37], [38], while inter-appendage expressed WNTs can activate non-canonical Wnt pathways (*e.g.* involving the kinase Jun) while inhibiting canonical signalling [31], [39]. Activation of the canonical Wnt pathway by pharmacological or genetic approaches induces formation of a broad range of epithelial appendages in vertebrates including hair follicles [37], [40], fungiform taste papilla [32], mammary glands [34], tooth [32], [41] and feathers [38], as well as tentacles in cnidarians [23], [42], [43]. To test the function of the canonical Wnt pathway in ostia formation in *Oscarella*, we thus activated it globally in cultured sponge pieces using two independent inhibitors of the negative regulator GSK3β: LiCl [44] and a newly-developed highly specific inhibitor called BIO [45]. We first confirmed that *O. lobularis* has conserved β-catenin and GSK3β genes, as in other sponges [16], [25]. 2–3 day treatments with LiCl or BIO provoked the development of ectopic ostia across the exopinacoderm surface, positioned between existing ostia (Figure 3D–E). Reminiscent of vertebrate skin appendages, ectopic ostia were first detectable as placode-like structures prior to epithelial invagination (Figure 3D–E).

**Figure 4:**
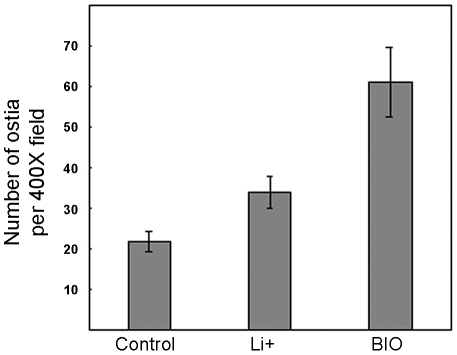
Quantification of the number of ostia in control sponge explants (n = 3) and in explants subjected to 48 h of 0.5 µm BIO (n = 3) or to 72 h (n = 3) of 40 mM LiCl. Error bars represent s.e.m. T-Student tests show significant differences between mean numbers of ostia in treated and control samples, (P<0.001 for both mean comparisons to control: 33.8±3.8 (mean±s.d, n = 5.) in the LiCl-treated samples, 61±8.6 (n = 3) in BIO-treated samples and 21.8±2.5 (n = 3) in control samples per 400× field. Thirty fields were counted for each.

The phenotypes observed using the two inhibitors were very similar; however BIO gave stronger and more rapid effects, in line with its higher specific activity. Statistical analyses confirmed that the number of ostia in treated sponges was significantly higher than in control sponges (Figure 4). The inhibitors also provoked other changes in morphology as revealed by SEM (Figure 3A–C), not unexpected since Wnt signalling is not only involved in initiating appendage formation but also in subsequent steps of morphogenesis [46], [47]. For example, similar changes in shape and size have been observed following lithium-treatment of taste papilla [36], mammary glands [34] and tooth [36] primordia.

Taken together our data indicate that canonical Wnt signalling has an early role in placode induction in *Oscarella* and probably in other aspects of ostia morphogenesis, as is the case in epithelial appendage morphogenesis in a variety of other species including cnidarians [43]. It thus appears clear that WNT/catenin and probably non-canonical Wnt signalling were involved in epithelial morphogenesis very early in animal evolution. Whether specific configurations of Wnt/β-catenin signalling and *Wnt* expression in epithelial patterning and morphogenesis have been conserved since the divergence of Homoscleromorpha and Eumetazoa or have appeared convergently remains open to debate. In any case, it appears clear that these two metazoan lineages use components from a common molecular toolkit to pattern epithelia, as they do to maintain epithelial undifferentiated cells [20]. More generally, *Oscarella lobularis*, with its true epithelium (pinacoderm) and underlying mesenchyme (mesohyl), should prove valuable to help evaluate the participation of epithelial patterning systems in the differentiation of body compartments [48] during animal body plan evolution.

## Materials and Methods

### Sequence data

Wnt sequences from *Oscarella lobularis* and *Amphimedon queenslandica* were identified by tBLASTn searches of our EST collection and genome trace archives (http://www.ncbi.nlm.nih.gov/Traces) respectively, based on similarity to vertebrate sequences. For *A. queenslandica*, sequences yielding tBLASTn values lower than 10^−3^ were retained, and the complete gene sequence reconstructed by retrieving overlapping sequence fragments. Gene structures (*i.e.* introns/exons) were determined using Genescan (http://genes.mit.edu/GENSCAN.html) and are available upon request. Primers and PCR conditions used for *β-Catenin* and *Gsk3-β* are listed in supplementary file S1 as well as accession numbers of all sequences.

### Alignment and phylogenetic analyses

Sequences were aligned by Clustal W (http://npsa-pbil.ibcp.fr/cgi-bin/npsa_automat.pl?page=/NPSA/npsa_clustalw.html). Available fragments of WNT sequence from the Homoscleromorph *Oscarella carmela* (Accession numbers: EB741370; EB741369; EB741367) were too short to be included in our data set.

Ambiguously aligned regions were removed using GBlock (http://www.phylogeny.fr/phylo_cgi/gblocks.cgi). The resulting alignment comprised 102 WNTs and 220 positions (available upon request).

Phylogenetic analyses were carried out by the maximum-likelihood (ML) method using the PhyML-2.4.4 program [49] and by Bayesian inference using the Mr Bayes-3.1.2 program [50]. The WAG [51] amino acid substitution model and a gamma distribution with four discrete categories were used.

For ML analysis, the gamma shape parameter and the proportion of invariant sites were optimized. Branch support was tested with bootstrapping (1000 replicates). For Bayesian analysis, six chains were run for 10,000,000 generations one of which was heated; after a burn-in of 2,500,000 generation every 100th tree was sampled only after posterior likelihoods reached stationary values.

### *In situ* hybridizations

RNA probes were synthesized with SP6/T7 DIG RNA Labelling Kits (Roche). Actin (positive control) antisense probes were transcribed from a clone of *Oscarella lobularis* after identification in an EST collection. As negative controls, sense probes were used in parallel with antisense probes in hybridization experiments.

*In situ* hybridization was performed on both whole fixed specimens (WMHIS) and histological sections mounted on slides.

Samples were fixed in 4% paraformaldehyde overnight. For sectioning, samples were embedded in paraffin. 10 µm sections were dewaxed, and all samples and sections rehydrated in PBS/alcohol with decreasing alcohol concentrations, followed by 10 min of 10 µg/mL Proteinase K treatment (for whole-mount samples) or 20 min in RIPA detergent treatment (for sections) (RIPA: 0.016 M NaCl; 0.02 M Tris base; 10^−3^ M EDTA; 0.1% SDS; 0.001% Igepal; 0.5% Na-deoxycholate). Samples and histological sections were post-fixed with 4% paraformaldehyde and acetylated by 0.25% anhydride acetic solution in triethanolamine 0.1 M. After washing in PBT, samples and sections were incubated in hybridization solution and then with a DIG-labelled probe (1 µg/ml) for 48 hours at 58°C. Hybridization solution: 50% deionised formamide, 5×SCC (pH 5), 1% SDS, 50 µg/ml yeast tRNA, 50 µg/ml heparin, Denhardt's 5× and 0.1% Tween 20. Subsequently, samples and sections were washed, at 58°C, in 50% formamide, SSC 5× pH 4.5 and then 50% formamide, SSC 2× pH 4. After blocking with 2% Blocking Reagent,1% Goat serum in MAB, both were reacted with anti-DIG antibody conjugated to alkaline phosphatase (dilution 1∶2000) overnight, in a humid chamber, at 16°C. After washing and equilibration to pH 9.5, they were incubated with BM purple, alkaline phosphatase substrate in the dark at 15°C for about 24 h.

### Treatment with GSK3β inhibitors

The pieces of *Oscarella lobularis* about 1 cm^3^ were cut *in situ*. The collection site was “la grotte d'Endoume” in Marseilles, a four meter deep underwater cave. They were left to heal during one day *in situ*. Then, they were cultivated in Petri dishes in roughly filtered sea water only (control), with 0.5 µM 6-bromo-indirubine-3′-oxime (BIO) [45] or with 40 mM lithium chloride (LiCl).

### Microscopy

For scanning electron microscopy (SEM) and semi-thin sections specimens were fixed in 2.5% glutaraldehyde in a mixture of 0.4 M cacodylate buffer and seawater (1∶4∶5; 1120 mOsm) and post-fixed in 2% OsO_4_, in seawater. Then they were dehydrated through a graded ethanol series. For SEM samples were critical point-dried, sputter-coated with gold-palladium, and observed under a Hitachi S570 SEM.

For semi-thin sections dehydrated samples were embedded in Araldite resin. The sections (0.5 µm of the thickness) were stained with toluidine blue.

### Statistical analyses

A two-tailed Student's t-test was used to assess the statistical significance of ostia numbers between treated and control samples. Thirty 400× fields were counted for each. A Shapiro-Wilk Normality Test confirmed that the three samples (Control, LiCl and BIO, n = 30) conformed to a normal distribution (P-value = 0.055, 0.126 and 0.979 respectively).

## Supporting Information

Supporting Information File S1 (1.70 MB DOC)
