# Supplementary material for: WNT/β-Catenin Signalling and Epithelial Patterning in the Homoscleromorph Sponge Oscarella
Source: PLoS One. 2009 Jun 8;4(6):e5823. doi: 10.1371/journal.pone.0005823 (PMC2688036; doi:10.1371/journal.pone.0005823)

**PCR conditions and primers for isolation of *β-catenin* and *gsk3β***

Degenerate primers pairs (see table below) were used as follows. 40 PCR cycles were used on cDNA. They were composed by: 40 cycles of denaturation at 94°C for 1 min, 60°C for 1 min and extension for 2 min at 72°C. MgCl_2_ concentration was 2mM, each primer was 0.4 µM concentrated.

Sequencing permitted to design specific primers (see table below) for 3’ RACE PCR. These primers were paired with *Not*-I(dT)18 (5’-AAC TGG AAG AAT TCG CGG CAG GAA TTTTTT TTT TTT TTT). 40 cycles of denaturation at 94°C for 1 min, 60°C for 1 min and extension for 2 min at 72°C. MgCl_2_ concentration was 1 mM, specific primer was 1 µM concentrated while *Not*-I(dT)18 was 0.2 µM concentrated.

|  | Primers sequence | |
| --- | --- | --- |
| *gsk3β* | Forward | 5’-GARYTNCARATHATG (ELQIM) |
|  | Reverse | 5’-YTCNGGNGCNCKRTARTA (YYRAPE) |
|  | RACE | 5’-CGGCCATACTGAAACTGTGC |
| *β-catenin* | Forward | 5’-GARGCNGARATGGCNCARAA(EAEMAQN) |
|  | Reverse | 5’-YTTRTCYTCNSWCATNCKRAA (FRMSDEK) |
|  | RACE | 5’-CCAAGTGGCCTCTTCTCAAG |

**List of Accession numbers :**

*Achaearanea tepidariorum :* Wnt16 (BAD12590) ; Wnt2 (BAD12591) ; Wnt5 (BAD12588) ; Wnt7 (BAD12589)

*Amphimedon queenslandica:* AqWntI (GQ144651); AqWntII (ABX90060) ; AqWntIII (GQ144650)

*Branchiostoma floridae :* Wnt1 (AAC80432) ; Wnt11 (AAF80555) ; Wnt3 (AAL37555) ; Wnt5 (AAL37556)

*Capitella sp*. (JGI identification number): Wnt1 (110406), Wnt2 (96953), Wnt4 (181867), Wnt5 (156046), Wnt6 (182518), Wnt7 (112156), Wnt8 (90169), Wnt9 (222661), Wnt10 (110385), Wnt11 (20087), WntA (216606), Wnt16 (149951);

*Cupiennius salei:* Wnt5 (CAC87041)

*Danio rerio:* Wnt2 (NP_571025); Wnt5 (NP_571012)

*Euprymna scoopes:* Wnt1 (ABD16194) ; Wnt4 (ABD16196)

*Gallus gallus:*  Wnt3 (XP_523686); Wnt4 (NP_990114); WNT6 (NP_001007595) ; Wnt7 (NP_989623) ; WNT8 (BAD95607) ; Wnt11 (P51891)

*Homo sapiens*: Wnt1 (NP_005421), Wnt2 (NP_003382), Wnt2B (NP_004176), Wnt3 (NP_110380), Wnt3A (NP_149122), Wnt4 (NP_110388), Wnt5A (NP_003383), Wnt5B (NP_116031), Wnt6 (NP_006513), Wnt7A (NP_004616), Wnt7B (NP_478679), Wnt8A (NP_490645), Wnt8B (NP_003384), Wnt9A (NP_003386), Wnt9B (NP_003387), Wnt10A (NP_079492), Wnt10B (NP_003385), Wnt11 (NP_004617), Wnt16 (NP_476509);

*Lottia gigantae* (JGI identification number): Wnt1 (170942), Wnt2 (139906), Wnt5 (130786), Wnt4 (130946), Wnt6 (136505), Wnt7 (179503), Wnt9 (180028), Wnt10 (136550), Wnt11 (109316), Wnt16 (105620), WntA (152125);

*Mus musculus* Wnt16 (AAI15812); Wnt9A (AAH66165)

*Nematostella vectensis*: Wnt1 (XP_001641494.1), Wnt2 (XP_001633773.1), Wnt3 (XP_001635899.1), Wnt4 (XP_001623100.1), Wnt5 (XP_001630693.1) Wnt6 (XP_001629451.1), Wnt7A (XP_001630717.1), Wnt7B (XP_001630717.1), Wnt8A (XP_001630032.1), Wnt8B (XP_001637576.1), Wnt10 (XP_001629450.1),Wnt11 (XP_001629964.1), Wnt16 (XP_001632885.1), WntA (XP_001637670.1)

*Oscarella lobularis :* OlWntI (GQ144646) ; OlWntII (GQ144647) ; Olbeta-catenin (GQ144649) ; OlGSK3 (GQ144648)

*Patella vulgata:* Wnt2 (CAD37171); Wnt10 (CAD37172)

*Platynereis dumerlii:* Wnt1 (CAD37164); Wnt4 (CAD37166) ; WNTA (CAD37169)

*Strongylocentrotus purpuratus*: Wnt1 (XP_780754.2), Wnt3 (XP_790595.1), Wnt4 (XP_786346.2), Wnt5 (XP_779946.1), Wnt6 (XP_790077.1), Wnt7 (XP_787051), Wnt8 (NP_999832.1), Wnt9 (XP_780823.2), Wnt10 (XP_781564.1), WntA (XP_797603.1);

*Takifugu rubripes:* Wnt8 (NP_001027726)

*Tribolium castaneum*): Wnt1 (XP_967980.1), Wnt5 (XP_974684.1), Wnt6 (XP_968055.1), Wnt7 (XP_973159.1), Wnt8 (XP_971439.1), Wnt10 (XP_968210.1), Wnt11 (XP_969261.1), WntA (XP_972893.1);

*Xenopus tropicalis* : Wnt3 (NP_001096552)

**Supplemental Figure**

Maximum Likelihood (Supplemental Figure 1) and Bayesian inference (Supplemental Figure 2) midpoint-rooted trees of the Wnt proteins. Clade support values are indicated to the right of nodes. Amq: *Amphimedon queenslandica*, Mm: mouse (*Mus musculus*), Bf: Amphioxus (*Branchiostoma floridae*), Nve: sea anemone (*Nematostella vectensis*), Pd: polychaete (*Platynereis dumerlii*), Pv: Gasteropoda (*Patella vulgata*), At: house spider (*Achaearanea tepidariorum*) , Spu: purple sea urchin (*Strongylocentrotus purpuratus*), Dr: zebrafish (*Danio rerio*), Cs: Arachnide (*Cupiennius salei*), Es: Cephalopoda (*Euprymna scoopes*), Gg: chicken (*Gallus gallus*), Hsa (*Homo sapiens*), Lgi: Gasteropoda (*Lottia gigantae*), Csp: Polychaeta (*Capitella sp*), Tca: Insecta (*Tribolium castaneum*),Tr: Teleostomi (*Takifugu rubripes*), Xt (*Xenopus tropicalis*).

Maximum Likelihood (Supplemental Figure 1)


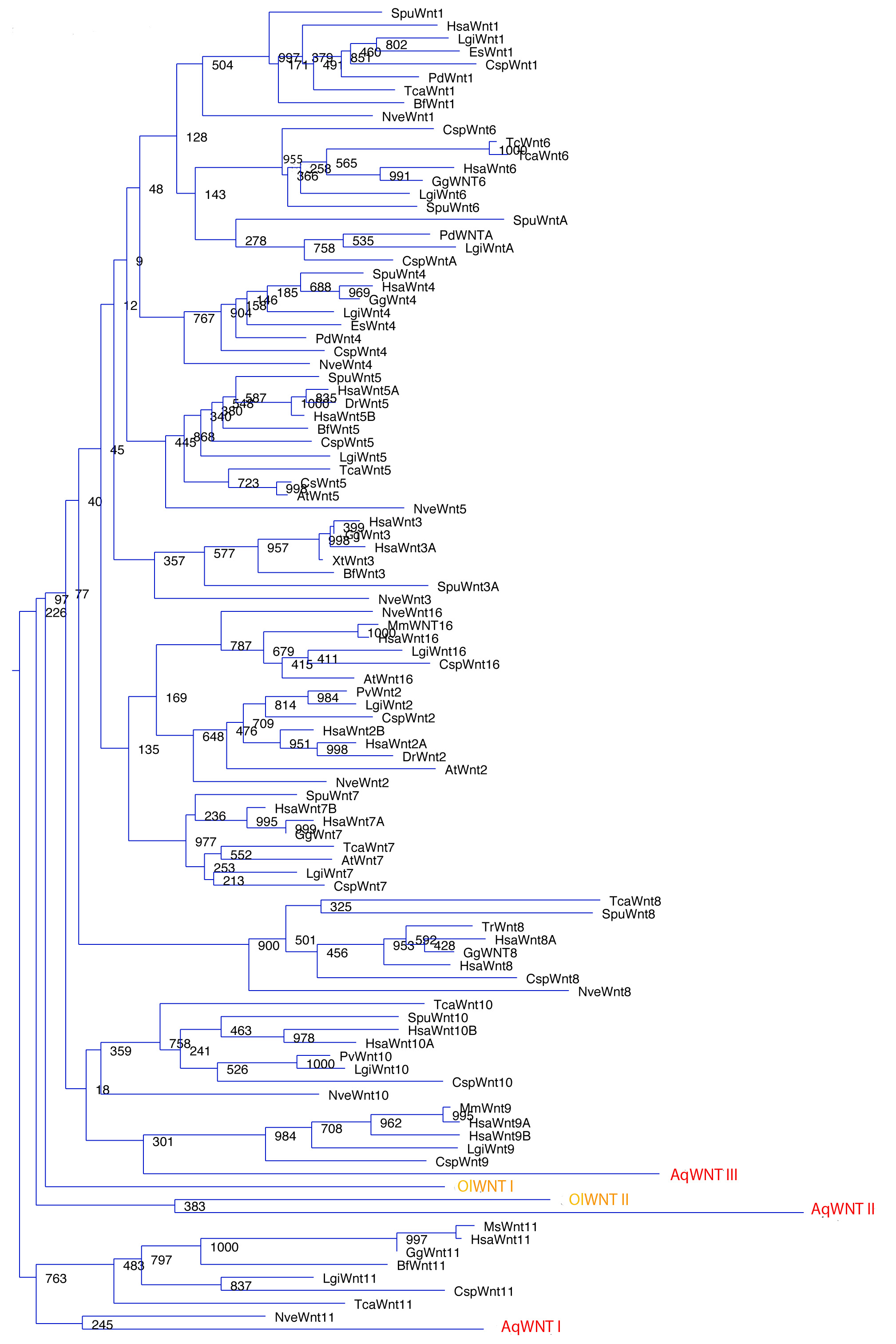


Bayesian Inference (Supplemental Figure 2)


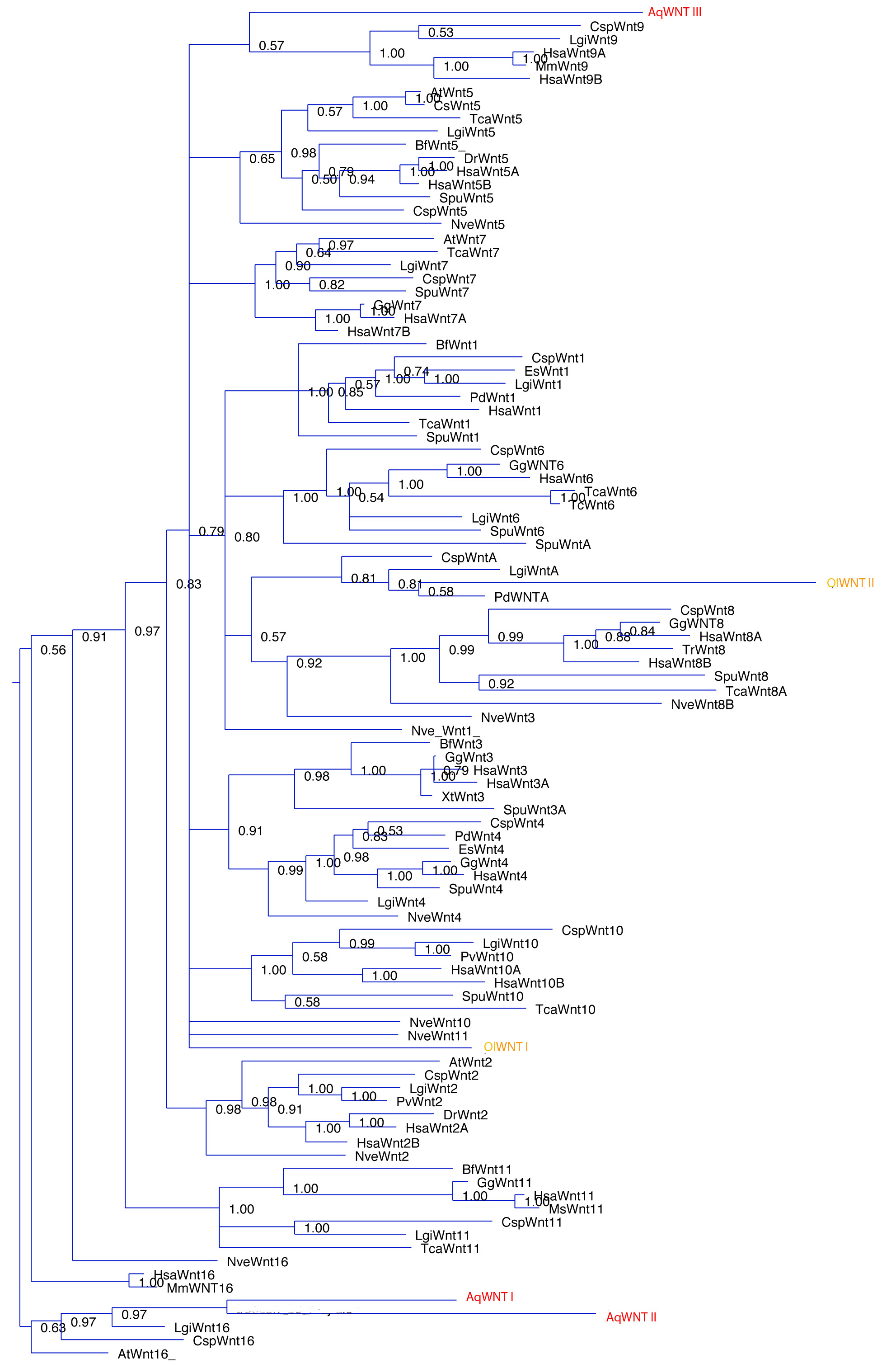

Supplement: Supporting Information File S1 — (1.70 MB DOC) [file pone.0005823.s001.doc]
